# Continuous pH monitoring using a sensor for the early detection of anastomotic leaks

**DOI:** 10.3389/fmedt.2023.1128460

**Published:** 2023-05-19

**Authors:** Michelle Huynh, Ricky Tjandra, Nour Helwa, Mohamed Okasha, Abdallah El-Falou, Youssef Helwa

**Affiliations:** ^1^ FluidAI Medical, Kitchener, ON, Canada; ^2^ Department of Mechanical and Mechatronics Engineering, University of Waterloo, Waterloo, ON, Canada; ^3^ Department of Chemistry, University of Waterloo, Waterloo, ON, Canada

**Keywords:** anastomotic leak, gastric leak, early detection, pH, PANI

## Abstract

Anastomotic leaks (AL) and staple line leaks are a serious post-operative complication that can develop following bariatric surgery. The delay in the onset of symptoms following a leak usually results in reactive diagnostics and treatment, leading to increased patient morbidity and mortality, and a clinical and economic burden on both the patient and the hospital. Despite support in literature for pH as a biomarker for early detection of AL, the current methods of pH detection require significant clinician involvement and resources. Presented here is a polyaniline (PANI)-based pH sensor that can be connected inline to surgical drains to continuously monitor peritoneal secretion in real time for homeostatic changes in pH. During this study, the baseline peritoneal fluid pH was measured in two pigs using the PANI sensor and verified using a benchtop pH probe. The PANI sensor was then utilized to continuously monitor the changes in the pH of peritoneal effluent, as a gastric leak was simulated. The inline sensors were able to detect the resulting local changes in drainage pH within 10 min of leak induction. The successful implementation of this sensor in clinical practice can both enable high efficiency continuous monitoring of patient status and drastically decrease the time required to detect AL, thus potentially decreasing the clinical and economic burden incurred by gastric leaks.

## 1. Introduction

Anastomotic leaks and staple line disruptions are considered the bane of postoperative complications following gastric and bariatric surgery (1). The reported incidence rate for postoperative leaks at gastrojejunostomies, enteroenterostomies, or at the gastroesophageal junction ranges from 1% to 5.6% (2, 3) depending on the type of bariatric surgery and is associated with up to 30% morbidity and mortality (4). In addition, AL can extend the patient length of stay to about 17 days, which is 15 days longer than the average length of stay for a non-leak patient (5) lowering patient quality of life. The development of AL incurs a clear clinical and economic burden on the patient and hospital. The current standard of care incites a diagnosis of AL upon symptom onset, which can range from 2 to 7 days postoperatively (6).

Since the clinical presentation of leaks may be subtle, delayed, or non-specific; the diagnosis of AL requires high levels of suspicion and careful monitoring of patients during their postoperative course (5). Symptoms indicative of AL include tachycardia, tachypnea, possible hypotension, persistent hiccup, fevers, chills, food intolerance, discomfort, nausea, vomiting, shoulder pain, abdominal pain, and hemodynamic instability (5–7). Leukocytosis and elevated C-reactive protein level are often noted as well. Imaging studies are usually conducted selectively based upon the patient's clinical progress. Contrast computed tomography (CT) scans with oral/IV contrast and/or an upper gastrointestinal (UGI) study with soluble contrast can be used to evaluate the integrity of anastomoses and detect leakage after bariatric surgery.

Despite being used frequently for definitive leak diagnosis, such studies have limitations and can delay the accurate diagnosis and management of leakage. It is reported that combined CT scans and diagnostic UGI studies carry a false-negative rate of 30% in AL patients (5, 8). Moreover, the time of diagnosis can vary between 0 and 28 days postoperatively (9). Delayed diagnosis contributes to increased hospital readmission rates, emergent procedures, and an increase in patient mortality (9). Currently, no optimal guideline exists for the proper management of AL after bariatric surgery. Therefore, management can vary and is highly dependent on the patient's overall hemodynamic status, size and location of the anastomotic insufficiency, and the extent of peritonitis/abdominal infection (10, 11). Management techniques include parenteral nutrition, antibiotic administration, CT-guided percutaneous drainage, endoscopic management (via clips, stenting, and fibrin glue), or surgical revision (10, 11). Therefore, there is an urgent need for technologies and techniques that can help clinicians detect and diagnose AL early (5).

Various postoperative clinical biomarkers have been identified in serum and peritoneal drain fluid to help with advanced detection of AL (12). Such biomarkers can minimize the sequelae associated with AL (including systemic inflammatory response syndrome, sepsis, and multiple organ failure) through early management. Some biomarkers outlined in research on gastrointestinal AL include pH, lactate, interleukin 6 (IL-6), interleukin 10 (IL-10), and tumor necrosis factor alpha (TNF*α*) (8, 11). Among these biomarkers, pH of peritoneal drain fluid was determined to be valuable as it is easy, quick and inexpensive to measure (12). It has been shown that a decrease in pH drainage fluid as early as postoperative day (POD) 1–3 can be used as an indicator of AL after gastrointestinal surgery (12–15).

Currently, many methods are employed in clinical settings to measure the pH of bodily fluids. The simple laboratory method includes collection of a sample, such as a urine sample, and conducting a laboratory test using a dipstick test, pH probe, or urine analyzers. A blood gas analyzer (BGA) can be utilized to obtain the pH of blood, in addition to various other biochemical properties. When conducting an assessment for gastroesophageal reflux disease (GERD), a Digitrapper™ pH-Z Testing System can be used. This involves passing a catheter into the esophagus through the nose. The measurements are recorded on a data logger which can be downloaded after the monitoring period and analyzed (16). To eliminate the presence of a catheter during monitoring, the Bravo® test was developed. This procedure requires temporary endoscopic placement of a small wireless pH capsule in the distal esophagus, which relays pH measurements to a recording device for 48 h (16, 17). The measurements can be visualized by a physician after completion of the monitoring period. Conventional gastric tonometry has been used for determining pH of gastric mucosa, through insertion of a modified nasogastric tube into the stomach, carrying a saline filled, gas permeable balloon at the tip. The tube is left inside to allow sufficient time for achieving equilibrium between the intraluminal fluid and saline, with regards to the pCO_2_, followed by collection of the saline and pCO_2_ assessment using BGA. The pH is then calculated using the Henderson-Hasselbalch equation (18).

Previous feasibility studies have presented evidence in favor of the implementation of a continuous patient monitoring system for AL detection in medical-surgical units (19) as it can decrease patient length of stay (20), allow for earlier administration of antibiotics, and lower the likelihood of readmission within 30 days of discharge (21). Data can also be streamed continuously to provide healthcare providers with a real-time postoperative state of the patient, potentially allowing for faster diagnosis of AL. Herein, a proof-of-concept continuous, inline monitoring system that is able to detect gastric leaks at their onset in porcine models is shown. The inline monitoring system uses a novel solid-state, potentiometric pH sensor system comprising of polyaniline (PANI), a pH-sensitive polymer, and a solid-state Ag/AgCl reference. The inline system attaches directly to prophylactic surgical drains (i.e., between the catheter and evacuator) such that all effluent comes in contact with the pH sensor prior to being collected in the evacuator.

Here, we demonstrate the utility of the PANI sensor in continuous bedside monitoring, through detection of pH changes in peritoneal effluent caused by simulated gastric leaks in a porcine model. This study also serves as a proof-of-concept for the integration of a pH sensor into prophylactic drains, which carries the potential to revolutionize postoperative patient monitoring.

## 2. Materials and methods

### 2.1. Materials

Jackson-Pratt (JP) flat silicon drains and 100 cc reservoirs (Cardinal Health, Ohio, USA) were used as a closed drainage system to suction fluid from the peritoneal cavity in porcine models. Flexible tubing (obtained by cutting the fenestrated end of the JP flat silicon drain) was used to connect the pumps and inline sensors. 5 cc syringes without needles were purchased from Terumo for peritoneal and gastric fluid aspiration (Terumo, Vaughan, Canada). Silicon wafers for the sensor dies were purchased from University Wafers (University Wafers, Boston, USA). The pH and temperature sensors were assembled in-house and sensor housings were 3D printed in-house using a Formlabs 3B SLA printer with Rigid resin (FluidAI Medical, Kitchener, Canada). A USB oscilloscope (Digilent Analog Discovery 2) and a custom-made data Acquisition (DAQ) board was used to interface with and log data from the pH sensor (Digilent Inc., Washington, USA). All pH measurements were also verified using a benchtop HANNA Instruments EDGE pH meter (HANNA Instruments, Quebec, Canada). A peristaltic pump was used in one of the experimental models to control flow from the peritoneum to the sensor (Cole-Parmer, Montreal, Canada). All electrochemical processes were done using an electrochemical workstation (Biologic SP-50, Tennessee, USA). Glass Ag/AgCl reference electrodes and Pt wire counter electrodes were purchased from CH Instruments (CH Instruments, Texas, USA). Thermistors and 10-position connectors were purchased from Digikey (Digikey Electronics, Minnesota, USA). A pure silver electrode (99.9%), hydrochloric acid (0.1 M), aniline (0.1 M), sulfuric acid (0.5 M), sodium phosphate monobasic (0.1 M) were purchased from Sigma-Aldrich and used as-is (Sigma-Aldrich, Missouri, USA). Potassium argentocyanide was purchased from Technic and used as-is (Technic Inc., Rhode Island, USA). Five-minute epoxy was purchased from a local retailer.

### 2.2. Sensor die patterning

The sensor dies were fabricated in the University of Waterloo's Quantum-Nano Fabrication and Characterization Facility (QNFCF) using standard photolithographic and deposition techniques (University of Waterloo, Waterloo, Canada). Multiple sensor dies were obtained from a single wafer. All traces and electrode pads were coated with high-purity gold and the rest of the wafer was insulated with silicon nitride. The surface area of the working and reference electrodes of the pH microsensors were measured at 0.85 mm^2^ and 1.7 mm^2^, respectively.

### 2.3. Solid state pseudo-reference electrode preparation

The wafers were first cleaned using DI water and isopropanol to remove any contamination from previous processes. Silver was electroplated onto the reference electrode with a constant current using a three electrode setup with the wafer as the working electrode, silver (99.9% purity) as the counter electrode, and a glass Ag/AgCl as reference electrode in a KAg(CN)_2_ solution. Similarly, chlorination of the electroplated silver was done using a constant current with platinum wire as the counter electrode and glass Ag/AgCl as a reference electrode in a 0.1 M HCl solution.

### 2.4. Electropolymerization of PANI

The wafers were first cleaned using DI water and isopropanol to remove any contamination from previous processes. PANI was electropolymerized onto the sensor die via cyclic voltammetry in a three-electrode setup with a platinum counter electrode and Ag/AgCl glass reference electrode in a solution of 0.1 M aniline and 0.5 M sulfuric acid. The potential was cycled between −0.15 V and 0.85 V vs. Ag/AgCl at a scan rate of 20 mV s–1 until the desired PANI thickness was reached.

### 2.5. Inline sensor assembly

**Figure 1:**
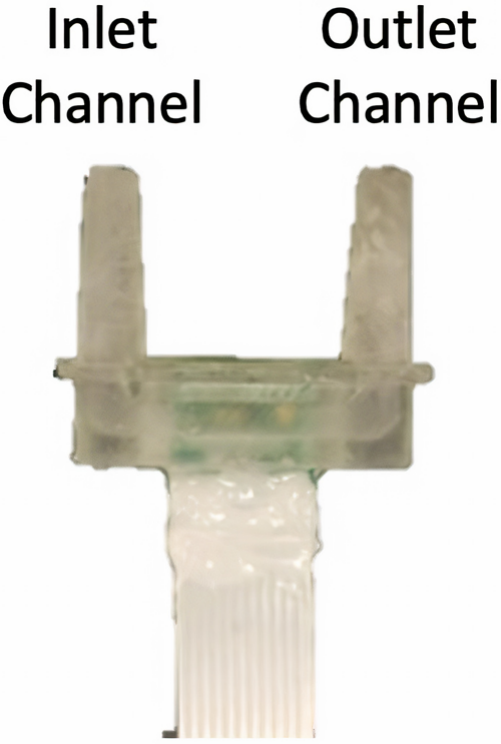
Fully fabricated inline sensor assembly.

After functionalization, the wafer was diced into individual dies. The temperature sensor (NTC thermistor) and 10-position FFC connector was then soldered onto the dies. The completed sensor was attached to the housing and encapsulated using a 5-minute epoxy (Figure 1).

### 2.6. Sensor calibration

Linear calibration curves for sensor output vs. pH were obtained using pH 6, 7, 8 buffers. The pH buffers were prepared in-house by dissolving 0.1M NaH_2_PO_4_ in DI water and titrating with KOH until the desired pH was reached. The calibration models can be found in Supplementary Materials (Supplementary Figure A1).

### 2.7. Surgical procedure and experimental protocol

Two Yorkshire pigs, designated as P-1 and P-2, were used in this prospective, acute animal study due to the anatomical and functional similarities between the GI tract of pigs and that of humans (22). The protocol for this study followed the guidelines of the Animal Welfare Act and was approved by the Institutional Animal Care Committee at the Li Ka Shing Knowledge Institute of St. Michael's Hospital.

On the day of surgery, both pigs were anesthetized through an intramuscular mix of ketamine at 20 mg/kg (6–7 ml) (Dechra Pharmaceuticals, Northwich, UK), xylazine at 2 mg/kg (3–3.5 ml) (Bayer, Mississauga, Canada), and atropine sulphate at 1 mg/25 kg (1–2 ml) (McKesson Corporation, Texas, USA). General anesthesia was maintained with 5% isoflurane (Fresenius Medical Care, Bad Homburg, Germany) delivered in oxygen for the duration of the procedure. The animals were monitored using jaw tone, pulse oximetry, body temperature, ETCO_2_ and ECG to ensure adequate anesthesia level and cardiopulmonary function. The skin on the anterior abdominal wall was prepared with a povidone-iodine solution (Rougier, Toronto, Canada) and draped in a sterile fashion. A 20 cm midline incision was then performed using the Bovie AARON 1,250 electrocautery generator (Bovie medical, Florida, USA), and a 5–10 ml peritoneal fluid sample was aspirated using an injection syringe from the right and left paracolic gutter of the peritoneal cavity. The pH of the peritoneal sample was measured using the inline sensor (see Supplementary Table A1 in Supplementary Materials) and verified with the HANNA benchtop meter. The benchtop meter was calibrated with a set of standard HANNA buffers (pH 4.0, 7.0, and 10.0) provided by the manufacturer before the surgical procedure (HANNA Instruments, Quebec, Canada).

**Figure 2:**
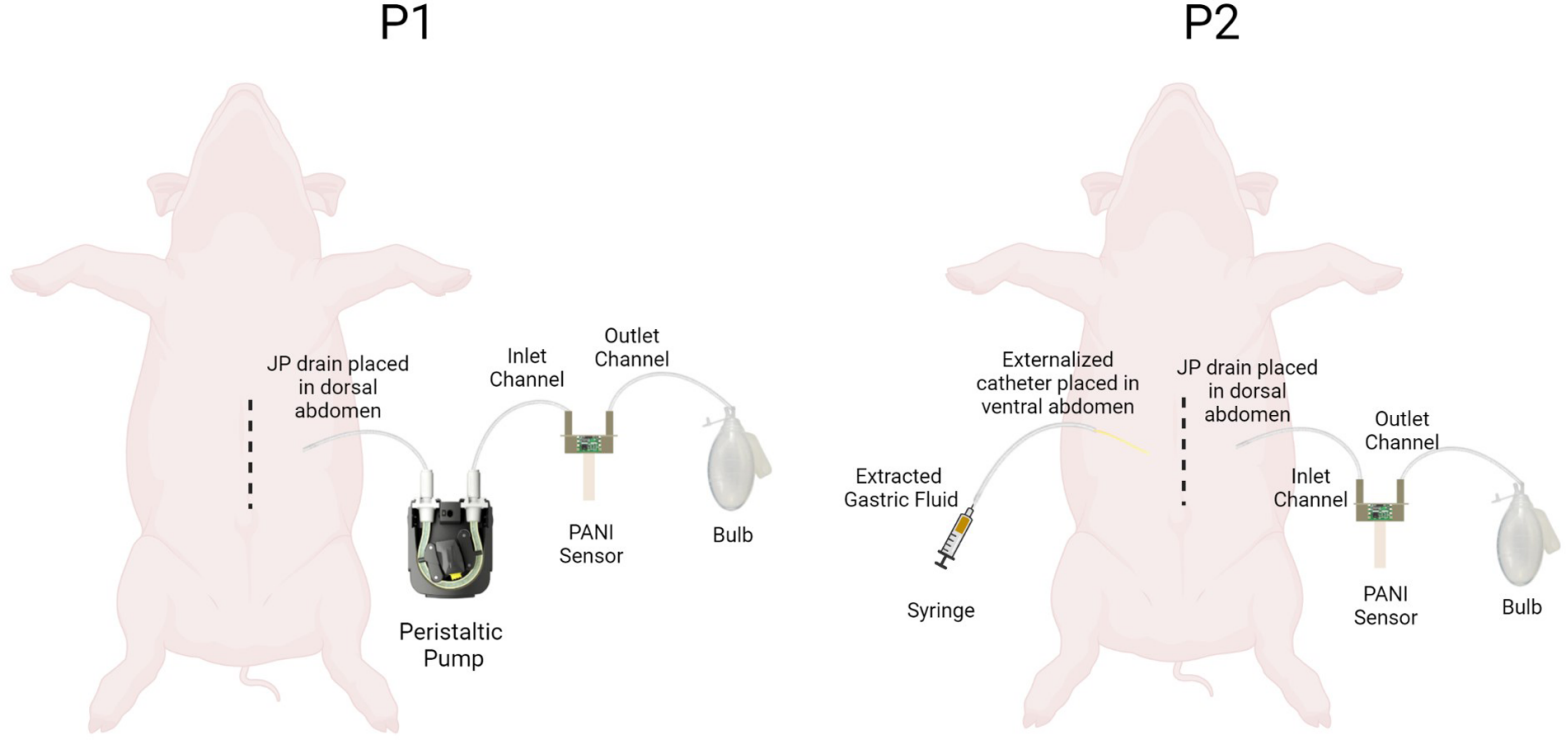
**Schematic showing pH measurement set up for P1 and P2.** The P1-S1 set up had a peristaltic pump attached proximal to the pH sensor. The P2-S2 set up had an additional externalized catheter to stimulate closed abdomen leaks.

For P-1 and P-2, the JP drain was positioned within the dorsal abdomen and brought out through the subcutaneous tissue exiting the abdominal wall at a point lateral to the midline incision. In P-1, the exteriorized tubing was connected to a peristaltic pump to facilitate constant drainage from the peritoneum. The sensor assembly (S-1) was setup such that the inlet and outlet of S-1 were connected to the peristaltic flow pump, and a 100cc bulb, respectively. To better simulate clinical application, P-2's sensor assembly (S-2) did not utilize a peristaltic pump. S-2 was positioned inline between the drainage end of the JP drain and the 100cc bulb. The applied negative pressure from the reservoir was used to facilitate drainage of peritoneal fluid through the sensor channel. In P-2, an additional catheter was positioned within the ventral abdomen and brought out through the subcutaneous tissue exiting the abdominal wall opposite to the JP drain to which S-2 was attached. The exteriorized catheter was occluded using a hemostat clamp and served as the designated point-of-entry for a 5cc syringe of gastric fluid used to simulate the closed-abdomen gastric leak. The set-up of S-1 and S-2 are depicted in Figure 2. S-1 and S-2 were then connected to a computer for data logging using the analogue discovery 2 (AD2) and DAQ board. For both P-1 and P-2, pre-leak baseline homeostatic measurements of the peritoneal fluid were collected using the PANI sensor to determine the normal physiological pH of peritoneal fluid in swine, prior to simulation of leaks.

Prior to leak induction 10 ml of gastric fluid was extracted for benchtop testing. This was done in both P-1 and P-2. Two techniques were utilized to simulate a gastric leak in this study: an open-abdominal and a closed-abdomen leak. An open-abdomen leak was performed whereby a 5 mm gastrotomy was created using a scalpel to represent the site of anastomotic leak. This was performed in P-1. A closed-abdomen leak was performed prior to the open-abdomen leak in P-2 by which a 10cc syringe with a 22-gauge needle was used to aspirate gastric fluid from the fundus. The midline incision was closed using running USP 1-0 Vicryl sutures after the administration of 5 mg/mL bupivacaine hydrochloride (Sterimax Inc., Oakville, Canada) though the abdominal incision to the subcutaneous tissue. Following closure, the gastric fluid was injected into the peritoneal cavity using the exteriorized catheter previously described. After approximately 15 min, following the passage of the gastric fluid through S-2, the stitches of midline incision were removed, and an open-abdomen leak was induced. The process of leak induction is summarized in Supplementary Figure A2. The pH of the peritoneal drainage fluid collected from P-1 and P-2 was measured using the inline sensor (see Supplementary Table A1 in Supplementary Materials) and verified with the HANNA benchtop meter.

Upon completion of data collection, P-1 and P-2 were administered a 200 mg/kg dose of pentobarbital sodium (Covetrus, Maine, USA) intravenously in the ear for euthanasia while under anesthesia.

### 2.8. Statistical analysis

pH data from both pigs were filtered with a first-order low pass filter on the DAQ board to reduce noise in sensor readings. All pH measurements obtained throughout the duration of this study were temperature-corrected based on the temperature readings measured by the on-chip thermistor. Numerous libraries on Python were used to analyze the data to assess the pH change upon leak induction. To characterize the baseline pH for both S-1 and S-2 descriptive statistics (mean ± standard deviation) were used. Leak detection by the sensors is defined as the point at which a peak pH change was observed in the oscilloscope timeseries. Time between leak detection by the sensors and the leak induction events was also calculated to evaluate the capacity of the sensor at detecting AL at onset.

## 3. Results

The baseline peritoneal drainage pH for S-1 and S-2 were 7.80 (s = 0.016) and 7.76 (s = 0.032), respectively. Leaks were characterized by a sharp increase or decrease in the real-time pH data measured by the inline sensors. Table 1 reports the point pH measurements surrounding each leak, the absolute change in pH between these points, and the sensor detection time. The baseline peritoneal and gastric fluid pH measurements obtained using the PANI sensor are reported in Supplementary Table A1.

**Table 1:** Extent of acute leak detection for all leak detection events observed in P-1 and P-2 models. The change in pH is reported for each leak event, as well as the detection time. *.*

Model	Leak event	Baseline pH	Peak pH	Absolute change in pH	Detection time (min)
P-1/S-1	Open-abdomen gastric	7.80	7.08	−0.72	4.93
P-2/S-2	Closed-abdomen gastric (Injection)	8.29	6.56	−1.72	3.73
	Open-abdomen gastric	8.04	7.56	−0.48	8.27

S-1 was connected inline to a peristaltic pump setup and was able to successfully detect the induction of a gastric leak within 4.93 min. S-2, which was connected inline to a drain with a terminal reservoir to reflect the sensor implementation in clinical settings, was able to detect the onset of a closed-abdomen leak in 3.73 min, and onset of an open abdominal gastric leak in 8.27 min. Gastric leak simulation detected by the sensors manifested as a decrease in pH sensor output due to the acidity of gastric fluid.

**Figure 3:**
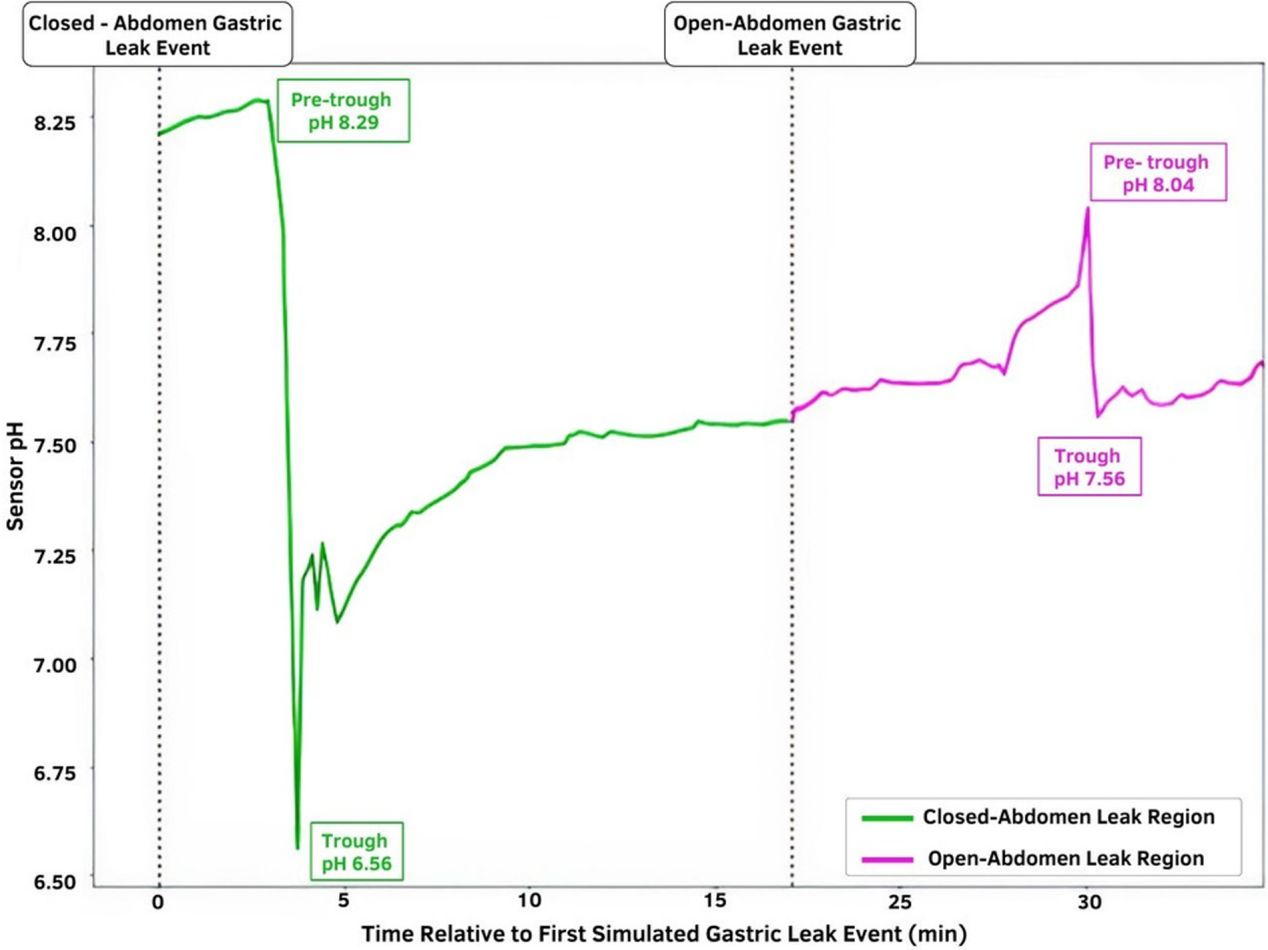
**Temporal pH sensor output with indicated leak induction events.** The green region represents the closed-abdomen gastric leak event pH in which gastric fluid was injected into the drain catheter; the magenta region represents the pH change observed after the open-abdomen gastric leak event. The time of leak induction is depicted by the dotted lines.

Figure 3 shows the continuous pH data gathered by S-2. The acute sensor responses for each leak event are indicated by the pre-trough and trough pH labels. The time of closed-abdomen leak induction was marked by the time at which 5 cc of gastric fluid was injected into the drain catheter. The open-abdomen leak label corresponds to the time at which the gastrotomy was created. Extremities of acute sensor response are also highlighted to indicate leak detection. The steep changes in pH following leak induction events are clearly visible in the real-time pH data.

## 4. Discussion

Anastomotic leaks and staple line disruptions are major postoperative complications that occur following gastric and bariatric surgery (1). The onset of an anastomotic leak can be devastating for patients leading to significant morbidity and mortality, need for additional diagnostic studies, potential reoperation, and extended length-of-stay (5). These outcomes amount to an average increase of $30,885 in hospital expenses for the patient, and an increase of $28.6 million per 1,000 AL patients for the involved hospitals (5, 23). The present study reports the use of real-time PANI-based inline sensors (S-1 and S-2) with existing surgical drains in two porcine models to demonstrate the successful detection of homeostatic imbalances in peritoneal drainage fluid pH following gastric leak simulation.

PANI is a desirable candidate for use in biosensors due to its linear potentiometric response to pH, stability over time, low synthesis cost (24, 25) and biocompatibility (26). The inline pH sensor reported here is non-invasive, can be readily incorporated into the current standard-of-care, and can be utilized to diagnose AL in real-time, significantly improving upon the current standard-of-care. In this study, sensors S-1 and S-2 detected the open-abdomen leak, and S-2 detected the closed-abdomen leak within minutes of leak simulation. The absolute change in pH following all leak induction events ranged from 0.48 to 1.72, showing clear responses to local changes in pH. This clearly highlights the value of using the PANI sensor for developing tools that target early detection of anastomotic leakage.

It is important to note that literature reports the pH of gastric fluid of pigs at 1.15 to 4.0 (27). This varies greatly from the measured values of peritoneal effluent pH in this study. The higher pH can be explained by the mixing of gastric fluid with peritoneal fluid prior to getting drained via the JP drain. Peritoneal fluid carries significant buffering capacity and has a pH in the range of 7.5 to 8.0 in humans (28). A study measuring the impact of various gases on peritoneal pH in pigs reported a baseline peritoneal pH of roughly 7.4 (29). Therefore, the PANI sensor measured a combination of peritoneal and gastric fluid, as opposed to only gastric fluid, which led to the high pH measurements observed. The baseline pH of gastric fluid was reported as 2.78 in P1 and 6.0 in P2 (Supplementary Table A1). Baseline pH for P1 falls within the expected range, while the higher baseline gastric fluid in P2 could be explained by the location of fluid extraction. The fluid was extracted from the base of the pylorus of the stomach, and the pH of the proximal small intestine is reported to be about 6.1 (30)

While these results are a powerful demonstration of the ability of the PANI sensor to rapidly detect changes in local pH caused by gastric leaks, limitations of the study should be noted. One limitation is that the study is restricted by its small sample size.

While the use of pH to detect AL has been reported previously in literature, to date the pH data in literature has been collected by manually taking samples of drainage fluid intermittently and analyzing it in the lab or by using gastric tonometry (13–15). These methods rely heavily on clinician involvement and the intervals between two measurements can be large. Intermittent testing limits the capacity of pH utility in future prognostic models for AL due to delays in data availability and added burden to the workflow of the care providers. The existing methods of continuous pH monitoring require invasive techniques for implanting sensors. Additionally, these methods have been developed to measure gastric and esophageal pH. Currently, there are no systems for use in continuous monitoring of pH obtained from abdominal drains. The inline PANI pH sensor mitigates these issues, as it can be readily attached to prophylactic drainage to provide a continuous stream of pH measurements, thereby conducting measurements in a non-invasive manner, with minimal input from medical personnel.

The utility of pH in this study is linked to the role of ischemia in development of AL, which refers to the inadequate supply of oxygen at the site of the anastomosis. Poor tissue perfusion leads to increased anaerobic metabolism, which produces lactate and carbon dioxide (both acidic in nature) as the byproducts, thereby decreasing pH (31). The onset of AL also triggers the infiltration and activation of immune cells in the tissue which manifests as inflammation. The increased cell activity results in greater oxygen demand, hence reducing local pH (31, 32). These principles have been utilized to assess pH as a biomarker for detection of colonic anastomotic leakage with great success (12–15). Given the widespread use of surgical drains across gastrointestinal surgeries—and the potential for anastomotic leaks in various part of the GI system—this technology may be applied in numerous contexts (e.g., colorectal, hepatobiliary, esophageal). It should be noted that processes that reduce the local acidity, such as proton pump inhibitors (PPI), would reduce the absolute changes noted in pH measurements. PPIs are weak bases used for suppression of gastric acid secretion (33). While their use would increase the pH of gastric acid, a drop in overall pH of peritoneal effluent is still expected due to the underlying ischemic and inflammatory processes, even though the absolute change may be lower.

In conclusion, the PANI-based pH sensor provides a strong candidate for use in tools designed for the early detection of anastomotic leakage. The sensor described here complements current standards of care, by harnessing widely used post-operative prophylactic drains for early detection of postoperative complications, including AL. Continuous patient monitoring of local pH can provide healthcare providers with quantitative measurements to assist clinical decision-making, as opposed to non-specific postoperative symptoms. Moreover, by providing a means of detecting and diagnosing leaks at earlier stage, patient outcomes can be significantly improved.

Further, the concept of integrating sensors for relevant biomarkers (such as pH) with surgical drains can be expanded to various other postoperative complications, beyond AL. Together, this data highlights the potential for use of inline sensors across surgical disciplines and contexts to develop tools that reduce the patient, healthcare, and economic burden of postoperative gastrointestinal complications.

## Data Availability

The raw data supporting the conclusions of this article will be made available by the authors, without undue reservation.
