# Supplementary material for: Continuous pH monitoring using a sensor for the early detection of anastomotic leaks
Source: Front Med Technol. 2023 May 19;5:1128460. doi: 10.3389/fmedt.2023.1128460 (PMC10235488; doi:10.3389/fmedt.2023.1128460)
Supplement: Supplementary file 1 [file Datasheet1.pdf]

Supplementary Material

Continuous pH Monitoring Using a Sensor for the Early Detection of Anastomotic Leaks

Michelle Huynh, Ricky Tjandra, Nour Helwa*, Mohamed Okasha, Abdallah El-Falou, Youssef Helwa

*** Correspondence:** Nour Helwa: nhelwa@fluidai.md

1 Supplementary Tables

Table A1: pH of control peritoneal drainage and gastric fluid samples measured with the inline sensors S-1 and S-2.

Model	Peritoneal pH	Gastric pH
**P-1/S-1**	7.80	2.78
**P-2/S-2**	7.76	6.0*

* The gastric fluid measurement is high since it was extracted from the pylorus at the base of the stomach. Typical pH of the proximal small intestine is reported to be about 6.1 (30).

2 Supplementary Figures

(a) (b)

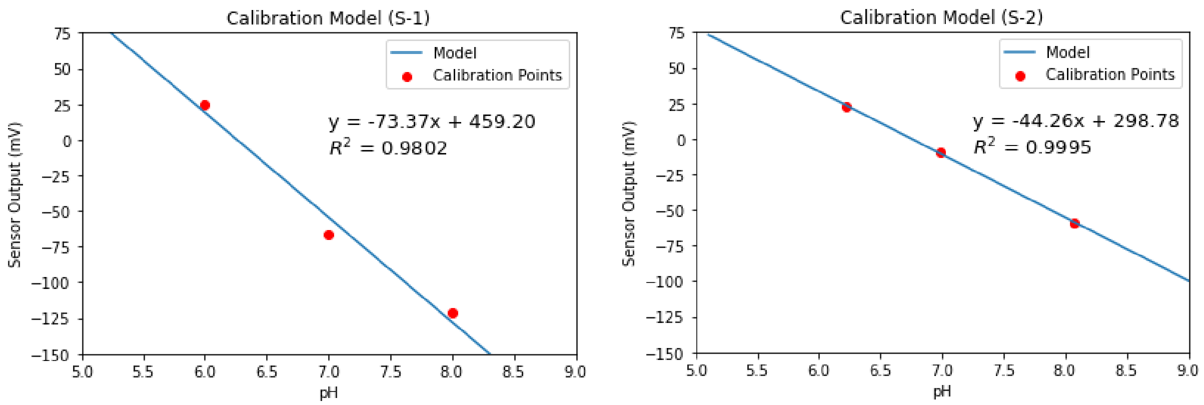

Figure A1: Calibration curve with regression equation and R^2 value for (a) S-1 and (b) S-2.

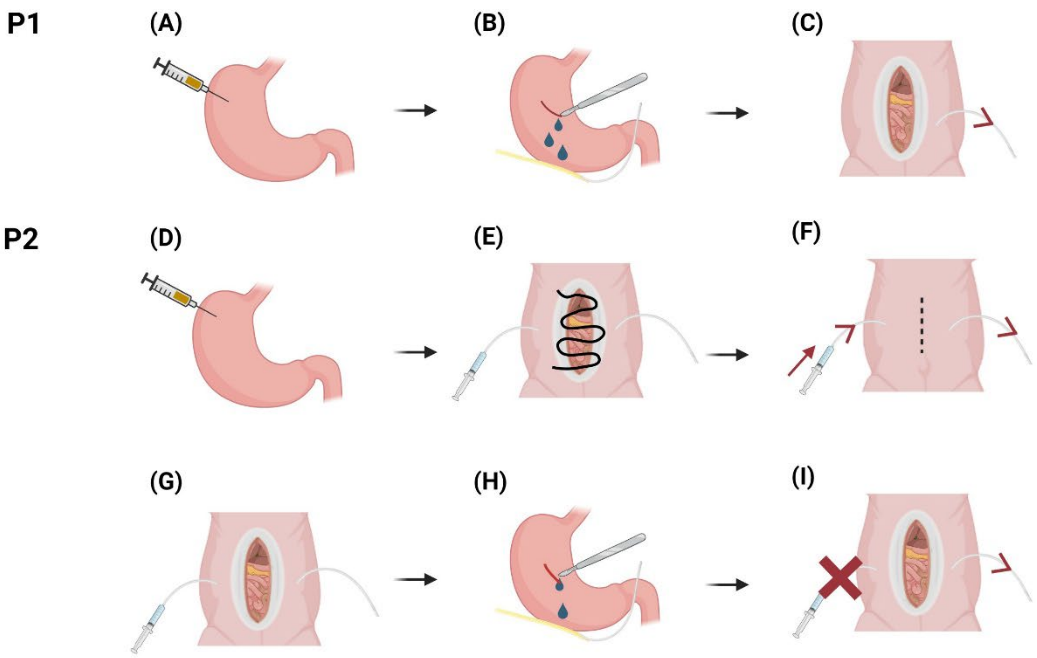

Figure A2: Schematic showing development of closed abdominal leak and open abdominal leak in P1 (A-C) and P2 (D-I).

(A) Extraction of gastric fluid for baseline assessment (B) creation of gastrotomy for open abdominal leak in P-1 (C) P-1 open abdominal leak monitoring using PANI sensor (D) gastric fluid extraction for baseline assessment and simulation of closed abdominal leak (E) midline incision closure for stimulating closed abdominal leak in P-2 (F) P-2 closed abdominal leak induction through externalized catheter and monitoring using PANI sensor (G) removal of midline incision stitches (H) creation of gastrotomy for subsequent open abdominal leak in P-2 (I) P2 open abdominal leak monitoring using PANI sensor
